# Clinical significance and biological functions of chemokine CXCL3 in head and neck squamous cell carcinoma

**DOI:** 10.1042/BSR20212403

**Published:** 2021-12-22

**Authors:** Jian Guan, Jinru Weng, Qiaosheng Ren, Chunbin Zhang, Liantao Hu, Wenjun Deng, Shizhen Lu, Xinyu Dong, Weidong Li, Yue Li, Weiqun Wang

**Affiliations:** 1 Department of Maxillofacial Surgery, Stomatological Hospital, Jiamusi University, Jiamusi 154002, Heilongjiang, China; 2 Basic Medical College, Jiamusi University, Jiamusi 154002, Heilongjiang, China; 3 Key Laboratory of Microecology-immune Regulatory Network and Related Diseases, Jiamusi 154002, Heilongjiang, China; 4 Department of Medical Technology, Zhang Zhou Health Vocational College, Collaborative Innovation Center for Translation Medical Testing and Application Technology Zhangzhou, Zhangzhou 363000, Fujian Province, China

**Keywords:** bioinformatics, CXCL3, HNSCC

## Abstract

CXCL3 plays extensive roles in tumorigenesis in various types of human cancers through its roles in tumor cell differentiation, invasion, and migration. However, the mechanisms of CXCL3 in head and neck squamous cell carcinoma (HNSCC) remain unclear. In our study, multiple databases were used to explore the expression level, prognostic value, and related mechanisms of CXCL3 in human HNSCC through bioinformatic methods. We also performed further experiments *in vivo* and *in vitro* to evaluate the expression of CXCL3 in a human head and neck tissue microarray and the underlying effect mechanisms of CXCL3 on the tumor biology of HNSCC tumor cells. The result showed that the expression level of CXCL3 in patients with HNSCC was significantly higher as compared with that in normal tissues (*P*<0.05). Kaplan–Meier survival analysis demonstrated that patients with high CXCL3 expression had a lower overall survival rate (*P*=0.038). CXCL3 was further identified as an independent prognostic factor for HNSCC patients by Cox regression analysis, and GSEA exhibited that several signaling pathways including Apoptosis, Toll-like receptor, Nod-like receptor, Jak-STAT, and MAPK signaling pathways may be involved in the tumorigenesis of HNSCC. CAL27 cells overexpressing or HNSCC cells treated with exogenous CXCL3 exhibited enhanced cell malignant behaviors, whereas down-regulating CXCL3 expression resulted in decreased malignant behaviors in HSC4 cells. In addition, CXCL3 may affect the expression of several genes, including ERK1/2, Bcl-2, Bax, STAT3, and NF-κB. In summary, our bioinformatics and experiment findings effectively suggest the information of CXCL3 expression, roles, and the potential regulatory network in HNSCC.

## Introduction

Head and neck squamous cell carcinoma (HNSCC) is one of the most common malignant tumors with a high mortality rate around the world. According to a recent study, there will be more than 500,000 new HNSCC patients in 2021, and it has become the sixth prevalent cancer worldwide [1]. HNSCC originates from the epithelial cells of the mucosal tissue of the upper aerodigestive tract, including the thyroid, larynx, and oral cavity [2]. Currently, the main treatment strategies for HNSCC include surgery, radiotherapy, and chemotherapy. Although significant progress has been made in traditional treatment methods for HNSCC, the clinical outcomes are still unsatisfactory due to the particularity of the anatomical parts of the head and neck. HNSCC not only directly affects the functions of breathing, eating, and language but also grows rapidly with the help of abundant blood supply and lymphatic reflux, and is prone to lymph node metastasis [3]. Although pathological diagnosis and TMN staging are still the main methods for evaluating the prognosis of HNSCC, it is completely insufficient because the existing indicators cannot fully cover all the patient information [4]. Therefore, it is of great important to distinguish the potential molecular markers for evaluating the prognosis and guiding clinical therapy of HNSCC.

Chemokines, a member of the cytokine subfamily, are known to play various important roles that are involved in many aspects of physiological and pathophysiological functions, including wound healing, inflammation, angiogenesis, and tumor metastasis. According to the arrangement of the amino-terminal (N-terminal) cysteines (Cys), chemokines are divided into different subfamilies: CXC (α), CC (β), CX3C (γ), and C (δ) [5]. In recent years, a growing number of studies indicated that CXC chemokine ligand (CXCL) 3 is constitutively expressed in several tumor types, and its high-level expression is extremely related to the advanced-stage, high-grade, and lymph node metastasis [6]. For example, it has been suggested that that CXCL3 was highly expressed in prostate cancer and high CXCL3 expression was closely related to the poor prognosis of patients [7]. Our recent study also reported that the protein expression level of CXCL3 in cervical cancer tissues was significantly higher than that in normal cervical tissues, and high CXCL3 level was positively correlated with high-risk stage of patients [8]. In addition to its constitutive expression, CXCL3 has been reported to be crucial for the initiation and progression of several human tumors by activating several signal pathways including MAPK/ERK [8], G-protein [9], and β-arrestin signaling pathways [10]. Our previous study has revealed that CXCL3 acts through its receptor (CXCR2) to activate the expression of the extracellular signal-regulated kinase (ERK) signaling pathway-related genes, including ERK1/2, Bcl-2, and Bax, thereby promoting the proliferation of cervical cancer cells through the MAPK/ERK pathway [11]. Recent research highlighted that CXCL3 appears to have a fundamental role in regulating neutrophil function through binding CXCR1/CXCR2 and activating G-protein and β-arrestin signaling pathways, thereby playing a crucial role in tumorigenesis [4]. Although many aspects of CXCL3 in several types of human cancers have been explored, its clinical significance and mechanism of action in HNSCC remain unclear.

In the present study, we evaluated the prognostic significance of CXCL3 gene expression in HNSCC through bioinformatic analysis of clinical features and survival information in public databases and analyzed the genomic alterations and functional networks associated with CXCL3 in HNSCC tissues. In addition, we also *in vitro* and *in vivo* explored the potential clinical significance and action mechanism of CXCL3 in HNCSS tissues and tumor cells, which may provide a novel idea for the future development in prevention, diagnosis and treatment of HNCSS.

## Materials and methods

### Data acquisition and bioinformatics analysis of CXCL3

The mRNA and clinical data of HNSCC samples were download from The Cancer Genome Atlas (TCGA) database (https://cancergenome.nih.gov). The independent data sets based on other HNSCC, including the Oncomine database (https://www.oncomine.org/), will be used for further verification. The patients with incomplete clinical information will be excluded. The chromosome location and gene structure of CXCL3 were obtained from the GeneCard database (https://www.genc ards.org/) and Ensembl database (http://asia.ensem bl.org/), respectively. We used the cBio Cancer Genomics Portal (http://cbioportal.org) to analyze the genetic alteration of CXCL3. The LinkedOmics database (http://www.linkedomics.org/) analyzed the differentially expressed genes related to CXCL3 in the TCGA HNSCC set. GSEA was performed to analyze CXCL3 in TCGA HNSCC set, with *P*<0.05 and false discovery rate (FDR) <0.25 as significantly enriched. To determine whether CXCL3 can become a new biomarker, we obtained the serum data of HNSCC patients from the GEO database (GSE183854, GSE39400) and analyzed the level of CXCL3.

### Clinical samples and immunohistochemistry

A commercial human head and neck tissue microarray, comprising 39 HNSCC and 19 normal head and neck tissues were obtained from bioaitech (cat. no.HN058Oc01; Shanxi, China) (Table 1). The clinical information of the samples is listed in Table 1. The microarray was baked in the oven at 60°C for 0.5 h followed by conventional deparaffinization and rehydration. After blocking endogenous catalase with 3% hydrogen peroxide, antigen retrieval was carried out in sodium citrate buffer (pH 6.0) at 100°C for 20 min. After that, the microarray was treated with goat normal serum (Boster, Wuhan, China) followed by further incubation with the CXCL3 antibody (ImmunoWay Biotechnology Company, U.S.A.) at 4°C overnight. After washing with phosphate-buffered saline (PBS) containing 5% Tween-20, the tissue microarray was subsequently treated with the second antibody, followed by incubation with a SABC kit (Boster, Wuhan, China) for 30 min at 37°C. Subsequently, color was developed with 3,3′-diaminobenzidine solution, and hematoxylin was applied for the nucleus counterstain at room temperature. The expression level of CXCL3 was calculated on the basis of the percentage and intensity of positive staining cells under an inverted microscope. The percentage of stained cells was ranked as follows: 0 = 0%, 1 = 5–25%, 2 = 26–50%, 3 = 51–75% and 4 = 76–100%. Whereas the intensity of staining cells was evaluated as follows: 0 = no staining, 1 = light yellow staining, 2 = brownish yellow staining, 3 = brown staining. The final staining grade was scored using the product of the two parameters for each sample: 0 = negative (one), 1–4 = weakly positive (+), 5–8 = positive (++), 9–12 = strongly positive (+++).

**Table 1:** Clinical sample information

Total no. of sample	58
Age (mean ± S.D)	52 ± 11
Gender	
Female	6
Male	52
Grade	
1	25
2	9
3	4
Stage	
I	0
II	6
III	14
IV	17
T	
1	0
2	23
3	9
4	5
Lymph node metastasis	
Y	29
N	10

### Quantitative real-time polymerase chain reaction

Total RNA was extracted from the HNSCC cell line by Trizolreagent (Sangon Biotech, Shanghai, China), prior to reverse transcription using a commercial kit (Tiangen, China), according to the manufacturer’s instructions. Quantitative real-time polymerase chain reaction (qRT-PCR) was performed with ABI7300 and the relative expression of CXCL3 was calculated based on the 2-ΔΔCT method. The forward and reverse primer sequences for CXCL3 were 5′-CGCCCAAACCGAAGTCAT-3′ and 5′-GTGCTCCCCTTGTTCAGTATCT-3′, and the primer sequences for β-actin were 5′-AGAAAATCTGGCACCACC-3′ and 5′-CTCCTTAATGTCACGCACGCACGA-3′, respectively.

### Cell culture and transfection

Human HNSCC cell lines (HSC4, CAL27 and KB) were purchased from ATCC. All cells were cultured in Dulbecco’s Modified Eagle’s medium (DMEM) with high glucose (GIBCO, U.S.A.) containing 10% fetal bovine serum (FBS, Biological Industries, Israel) and 100 U/ml penicillin/streptomycin (GIBCO, U.S.A.) at 37°C in a humidified atmosphere of 5% CO2. The lentiviral vectors containing human CXCL3 gene and siRNA sequences targeting human CXCL3 gene were constructed, followed by producing the lentiviral particles carrying these vectors by GeneCopoeia Co., Ltd. (Guangzhou, China). HSC4 and CAL27 cells were seeded in a 24-well plate at 5 × 10^4^ per well, and when the cell confluence reached 50%, the culture medium was replaced with lentiviral particle-containing culture medium for cell transfection to obtain Si-CXCL3 HSC4 cells (HSC4 Si-CXCL3) and their negative control cells (HSC4 Si-NC), CXCL3-overexpressing CAL27 cells (CAL27 O-CXCL3) and their negative control cells (CAL27 O-NC). The transfection efficiency was identified by qRT-PCR.

### Cell proliferation assay

We used the following two experiments to determine the role of CXCL3 in tumor cell proliferation: (1) Exogenous experiment: The HNSCC cells including HSC4, KB, and CAL27 were, respectively, seeded in 96-well plates with 2 × 10^3^ cells per well containing 100 µl of complete medium with different concentrations of exogenous recombinant human CXCL3 (PeproTech). (2) Autocrine experiment: The transfected HSC4 and CAL27 cells were seeded in 96-well plates with 2 × 10^3^ cells per well containing 100 µl of complete medium, respectively. After 24, 48, or 72 h of culture, 10 µl of CCK-8 reagent was added into each well and the 96-well plates continued to be incubated in a humidified incubator for 1 h, and the optical density (OD) for each well was finally detected at 450 nm with a microplate reader (BioTek Instruments, Inc.).

### Cell migration assay

The 24‐well transwell chambers (Corning) were performed to analysis the activity of tumor cell migration. In agreement with proliferation assay, the parental and transfected HNSCC cells were employed for exogenous and autocrine experiments, respectively. (1) Exogenous experiment: A total of 2 × 10^4^ HSC-4 cells were seed in upper chamber with serum-free medium, and the medium containing 20% FBS and various concentrations of exogenous recombinant human CXCL3 (PeproTech) was added into the bottom chamber. (2) Autocrine experiment: The transfected HSC4 and CAL27 cells in serum-free medium were respectively added into the upper chamber at 2 × 10^4^ per well, and the medium containing 20% FBS was placed in the bottom chamber. After 48 h of incubation, the upper chamber cells were wiped with a cotton swab, and the chambers were fixed with ethanol for 20 min, followed by stain with 0.1% Crystal Violet for 30 min. Subsequently, the number of cells that passing through the chamber was recorded under an inverted microscope.

### Clonogenic ability assay

The transfected HSC4 and CAL27 cells were seeded in 500 μl medium containing 10% FBS at 5 × 10^2^ per well, respectively. The culture medium was replaced with fresh medium every 3–4 days. After incubation in a 37°C humid atmosphere for 14 days, the cell clones were stained with 0.01% Crystal Violet at room temperature for 15 min. Clonogenic ability of tumor cells was scored as followed: Clone formation rate = the number of clones/the number of inoculated cells × 100%.

### Wound healing assay

The transfected HSC4 and CAL27 cells were seeded into 6-well plates with 3 × 10^5^ per well. When the cell reached 90% confluence, a sterile tip was used to create wound by vertical scraping the monolayer cells on the bottom of plates. After washing with PBS, the cells were further incubated with serum-free medium for 24 h. The wound was digitally photographed on 0 and 24 h, respectively, and cell migration area was exploited to determine wound healing status.

### Flow cytometry

The transfected HSC4 and CAL27 cells were seeded in 6-well plates with 3 × 10^5^ per well containing 1 ml DMEM/high glucose medium supplemented with 10% FBS. After incubating for 12 h, cells were fixed with 70% pre-chilled ethanol at 4°C for 12 h. PI/RNase Staining Buffer (Becton, Dickinson and Company, U.S.A.) and AnnexinV-FITC (Becton, Dickinson and Company, U.S.A.) were used to detect apoptosis by flow cytometry, according to the manufacturer’s instructions.

### Western blotting

Total cell protein was extracted using RIPA lysis buffer (cat. no. P0013C; Beyotime Institute of Biotechnology). Following measuring protein concentration using a BCA protein concentration determination kit (cat. no. AR0197, Wuhan Boster Biological Technology, Ltd.), the protein was separated on an 8% or 12% SDS-PAGE gel, and further blotted onto a PVDF membrane. After blocking the transferred imprinted PVDF membrane with 5% skimmed milk in TBST at 37°C for 60 min, the membrane was incubated with the primary antibody overnight at 4°C. The main antibodies were as follows: β-actin (1:5000; cat. no. AB0011; Shanghai Abways Biotechnology Co., Ltd.); Bax (1:1000; cat. no. AY0553; Shanghai Abways Biotechnology Co., Ltd.); Bcl-2 (1:1000; cat. no. AB3359; Shanghai Abways Biotechnology Co., Ltd.); STAT3 (1:1000; cat. no. CY5165; Shanghai Abways Biotechnology Co., Ltd.); phosphorylated (p)-STAT3 (1:1000; cat. no. CY5291; Shanghai Abways Biotechnology Co., Ltd.); ERK (1:1000; cat. no. CY5487; Shanghai Abways Biotechnology Co., Ltd.); phosphorylated (p)-ERK (1:1000; cat. no. CY5277; Shanghai Abways Biotechnology Co., Ltd.); phosphorylated (p)-AKT (1:1000; cat. no. CY5277; Shanghai Abways Biotechnology Co., Ltd.); NF-kappaB p65 (1:1000; cat. no. CY2329; Shanghai Abways Biotechnology Co., Ltd.). On the next day, the membrane was incubated with HRP-conjugated goat anti-rabbit or rabbit anti-mouse secondary antibody, (1:5000; cat. nos. BA1056 and BA1058, respectively; Wuhan Boster Biological Technology, Ltd.). Finally, a ECL kit (cat. no. P0018FS; Beyotime Institute of Biotechnology) was used to visualize the immune protein blot.

### Statistical analysis

GraphPad Prism 8.0 software and R software were used to draw related graphics. IBM SPSS23.0 and R software were used for statistical analysis, including COX regression analysis, two independent sample *T*-test, Wilcoxon rank-sum test, etc. *P*-value (two-sided test) less than 0.05 is considered significant.

## Result

### Structure and expression of CXCL3 in HNSCC

**Figure 1:**
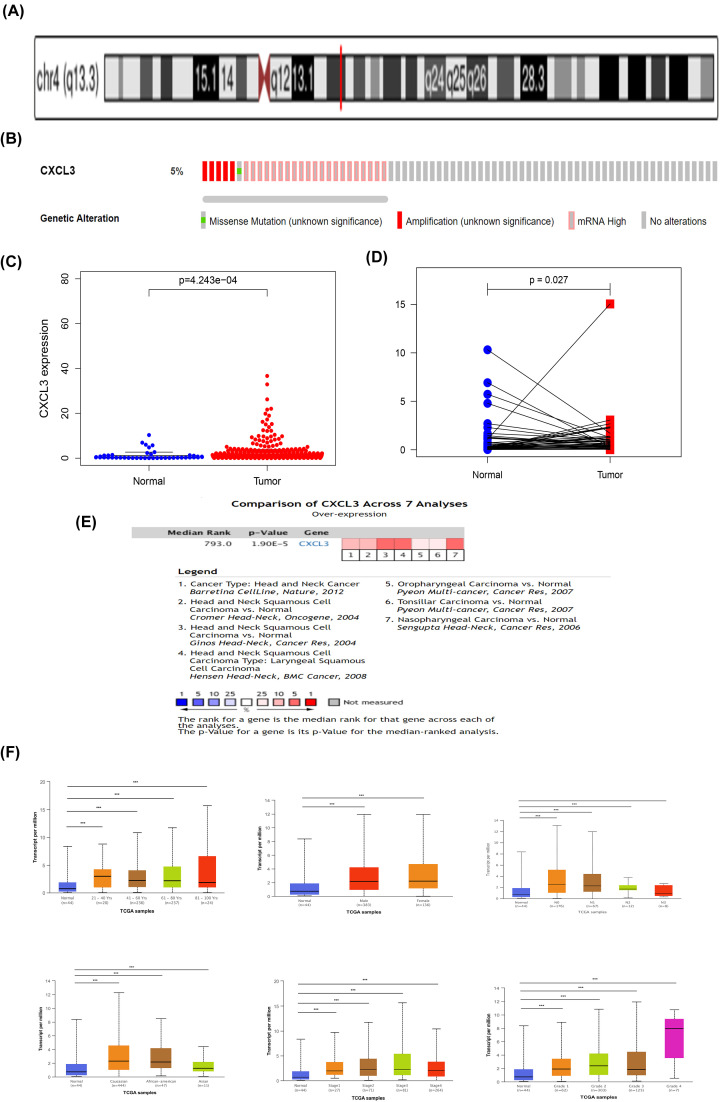
Gene structure, expression level and clinical analysis of CXCL3 in HNSCC (**A**) Chromosome localization and gene structure of CXCL3 in human. (**B**) The alterations of CXCL3 in HNSCC. (**C**) CXCL3 expression in normal and tumor tissues. (**D**) CXCL3 expression in paired tissues. (**E**) CXCL3 is over-expression (red) in HNSCC by Oncomine meta-analysis comparing with normal tissue. (**F**) Boxplot showing relative expression of CXCL3 in subgroups of patients with HNSCC, stratified based on gender, age and other criteria; *P<0.05; **P<0.01; ***P<0.001; HNSCC, head and neck squamous cell carcinoma.

CXCL3 is located at 4q21, and consists of 4 exons and 3 introns (Figure 1A). We used the sequencing data of the TCGA HNSCC set in the cBioPortal database to determine the genomic alterations of CXCL3 (Figure 1B). These alterations mainly occurred in mRNA High, amplification, and missense mutation. We first evaluated the mRNA expression of CXCL3 in HNSCC from the TCGA database and found that the expression level of CXCL3 in tumor tissues was significantly higher than that in normal tissues (Figure 1C). The expression in paired HNSC tissues is also significantly higher than that in adjacent normal tissues (Figure 1D). At the same time, data in Oncomine database verified that the expression of CXCL3 was significantly higher in HNCC tissues than in normal tissues (Figure 1E). The transcription level of CXCL3 was significantly higher in HNCC patients than healthy people in subgroup analyses based on gender, age, disease stages and tumor grade (Figure 1F). In addition, data from the GEO database further confirmed that the expression of CXCL3 in the serum of HNSCC patients was higher than that of normal people (Supplementary Figure S2). Therefore, the expression of CXCL3 is crucial to the prognosis of HNSCC.

### CXCL3 is an independent prognostic factor for overall survival

**Figure 2:**
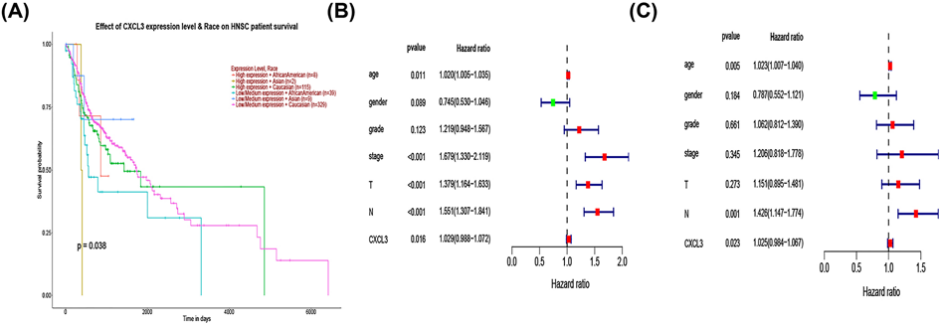
Kaplan–Meier analysis, and univariate and multivariate Cox regression analyses of clinical characteristics associated with overall survival of HNSCC (**A**) Kaplan–Meier curve for overall survival in HNSCC. (**B**) Forest plot of the univariate Cox regression analysis in HNSCC. (**C**) Forest plot of the multivariate Cox regression analysis in HNSCC; HNSCC, head and neck squamous cell carcinoma.

Kaplan–Meier survival analysis showed that patients with high expression of CXCL3 in different races have a poor overall survival (*P*<0.38) (Figure 2A). Univariate COX analysis showed that CXCL3 was significantly associated with poor prognosis (hazard ratio [HR] = 1.029, 95% CI = 0.988–1.072, *P*=0.016) (Figure 2B). Multivariate Cox analysis also verified that the high expression of CXCL3 is an independent prognostic factor affecting the survival of HNSCC patients (HR = 1.025, 95% CI = 0.984–1.067, *P*=0.023) (Figure 2C).

### Enrichment analyses of co-expression genes correlated with CXCL3 in HNSCC

**Figure 3:**
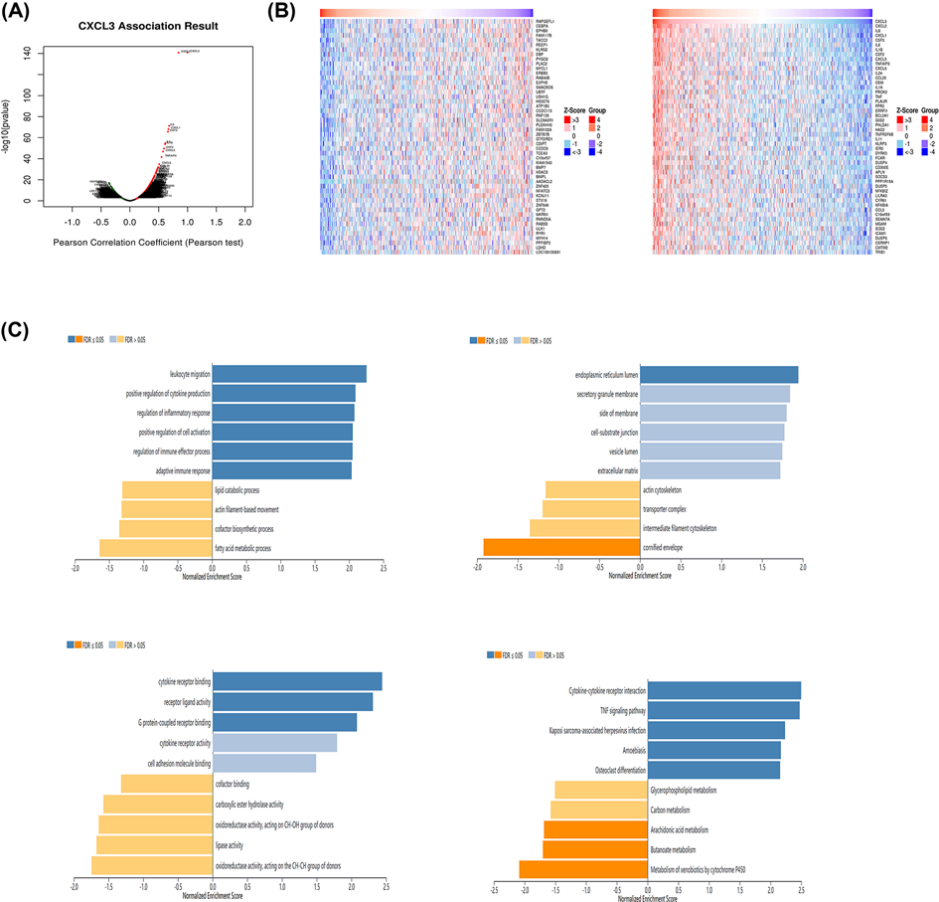
Genes differentially expressed in correlation with CXCL3 in HNSCC (LinkedOmics) (**A**) A Pearson test was used to analyze correlations between CXCL3 and genes differentially expressed in HNSCC. (**B**) Heat maps showing genes positively and negatively correlated with CXCL3 in HNSCC (TOP 50). (**C**) Significantly enriched GO annotations and KEGG pathways of CXCL3 in HNSCC; HNSCC, head and neck squamous cell carcinoma.

We used the LinkedOmics database to analyze the mRNA sequencing data of the TCGA HNSCC set. The results showed that 2041 genes (green) were significantly negatively correlated with CXCL3, and 2535 genes (red) were significantly positively correlated with CXCL3 (FDR<0.05) (Figure 3A). The 50 significant genes correlated with CXCL3 (Figure 3B). This result showed a widespread effect of CXCL3 on the transcriptome. GO analysis showed that the differentially expressed genes related to CXCL3 are mainly involved in leukocyte migration and endoplasmic reticulum lumen and cytokine receptor binding. KEGG pathway analysis shows that enrichment in the cytokine–cytokine receptor interaction, TNF signaling pathway, and Kaposi sarcoma-associated herpesvirus infection (Figure 3C).

### GSEA analysis of CXCL3 related signal pathways in HNSCC

**Figure 4:**
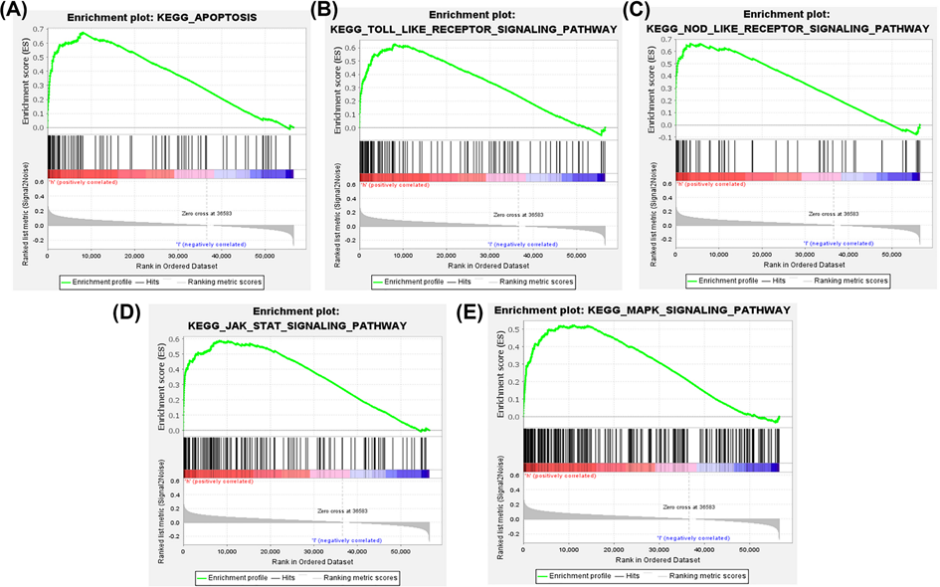
Enrichment plots from GSEA Apoptosis (**A**), Toll-like receptor (**B**), Nod-like receptor (**C**), Jak-STAT (**D**), and MAPK (**E**) signaling pathways may be involved in the tumorigenesis of HNSCC in response to CXCL3.

GSEA analysis was used to perform KEGG analysis on the CXCL3 gene in the TCGA HNSCC set, with *P*<0.05, FDR<0.25 as the standard, and ranking in descending order of enrichment value from high to low, and selecting the most enriched signal pathway. Highly expressed CXCL3 gene is highly enriched in Apoptosis, Toll-like receptor signaling pathway, Nod-like receptor signaling pathway, Jak-STAT signaling pathway, and MAPK signaling pathway (Figure 4).

### Exogenous administration of CXCL3 contributes to HNSCC cells malignant behaviors

**Figure 5:**
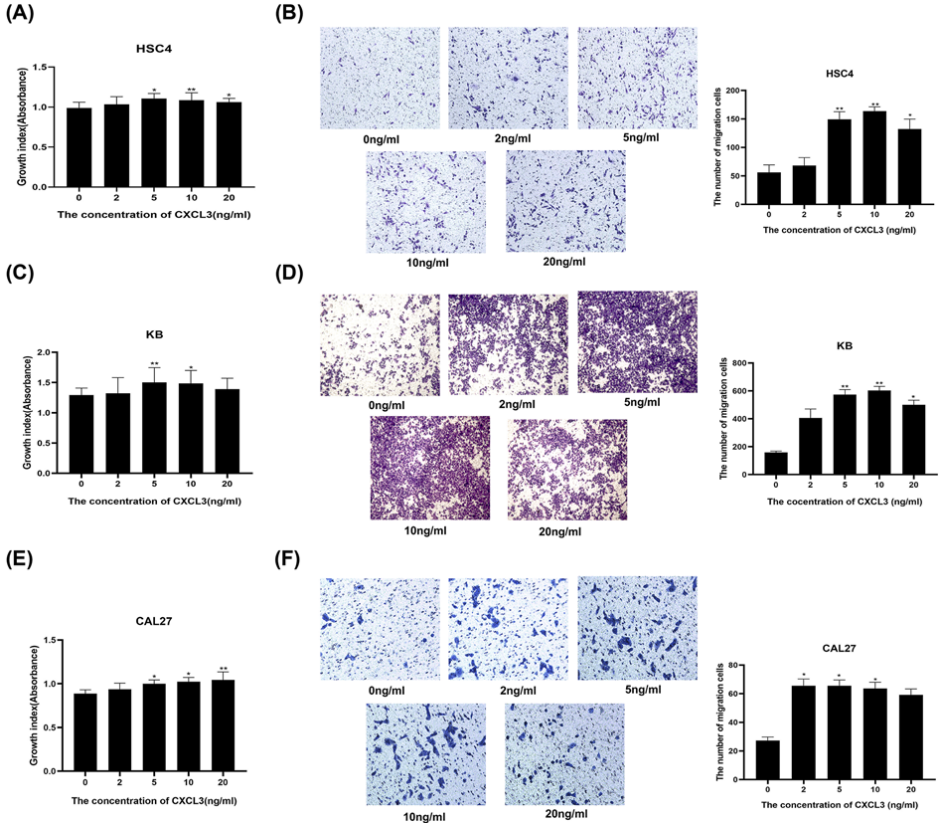
The roles of exogenous CXCL3 in HNSCC cell proliferation and migration (**A**,**C,E**) Representative images and boxplot of Transwell analysis for HNSCC cells treated with different concentrations of exogenous CXCL3. (**B,D,F**) CCK‐8 analysis of HNSCC cells treated with different concentrations of exogenous CXCL3; *P<0.05; **P<0.01; HNSCC, head and neck squamous cell carcinoma.

To further understand the effect of CXCL3 on the malignant behaviors of HNSCC cells, we used different concentrations of recombinant human CXCL3 to treat HSC4, KB, and CAL27 cells. The results showed that compared with the 0 ng/ml group, the proliferation and migration abilities of HSC4 cells in the 5, 10, and 20 ng/ml groups were significantly enhanced (Figure 5A,B). The proliferation abilities of KB cells in the 5 and 10 ng/ml groups, and the migration abilities of KB cells in the 5, 10, and 20 ng/ml groups were significantly enhanced (Figure 5C,D). Futhermore, the proliferation abilities of CAL27 cells in the 5, 10, and 20 ng/ml groups, and the migration abilities of CAL27 cells in the 2, 5, and 10 ng/ml groups were significantly enhanced (Figure 5E,F).

### CXCL3 is overexpressed in HNSCC tissues and cell lines

**Figure 6:**
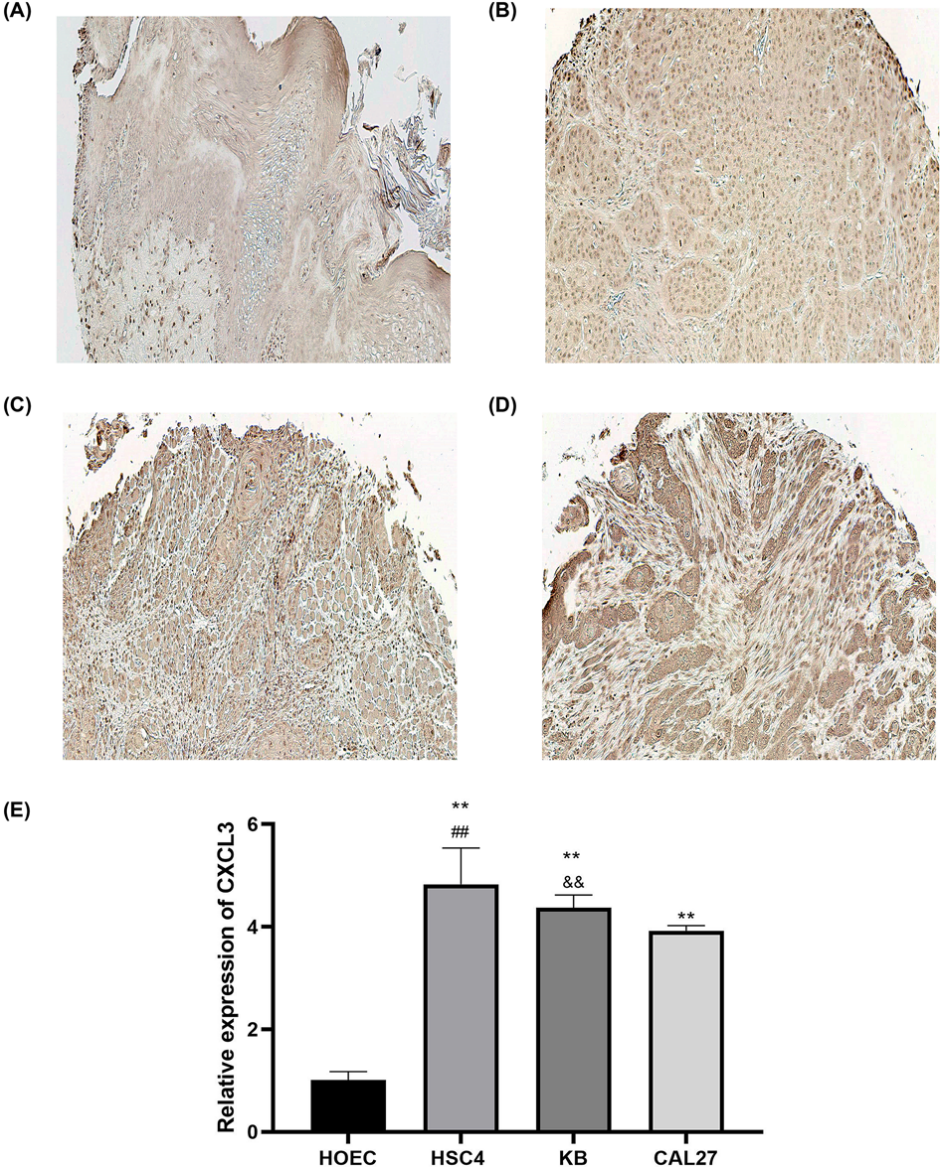
CXCL3 expression in human HNSCC tissues and cell lines (**A**) Representative image from normal head and neck tissue with weak staining intensity of CXCL3. (**B–D**) Representative images from Stage I, II, and III HNSCC tissue samples exhibiting strong staining intensity of CXCL3. (**E**) CXCL3 mRNA expression levels in HOEC, HSC4, KB, and CAL27 by qRT-PCR. **P<0.01 vs. HOEC cells; ^##^*P*<0.01 vs. KB and CAL27 cells; ^&&^*P*<0.01 vs. CAL27 cells; HNSCC, head and neck squamous cell carcinoma.

To analyze the clinical significance of CXCL3 in HNSCC, we evaluated the expression of CXCL3 through IHC analysis in a tissue microarray (Figure 6A–D). The IHC score showed that the expression level of CXCL3 in HNSCC tissues was significantly higher than that in normal tissues (Table 2). In addition, the up-regulated expression of CXCL3 was positively related to the high-stage but not related to other pathological parameters (Table 2). We also detected the expression of CXCL3 in three HNSCC cell lines (HSC4, KB, and CAL27) and one normal oral epithelial cell line (HOEC) by qRT-PCR, and the results showed that the mRNA expression level of CXCL3 was significantly higher in HSC4, KB, and CAL27 cells than that in HOEC cells (Figure 6E). At the same time, the CXCL3 expression level in HSC4 cells was higher than KB and CAL27 cells, and the CXCL3 expression level in KB cells was higher than CAL27 cells (Figure 6E). Therefore, HSC4 and CAL27 were recruited for subsequent studies.

**Table 2:** Clinical characteristics and immunohistochemical results of oral tissue microarray

Clinical characteristic	*n*	The score of CXCL3	*P*-value
Tissue			<0.01
Normal	19	11.06 ± 1.66	
Tumor	39	19.82 ± 2.48	
Age			=0.476
≥55	22	16.51 ± 2.13	
<55	17	16.10 ± 1.41	
Tumor size			=0.643
≥3.5 cm	26	16.58 ± 1.75	
<3.5 cm	12	16.28 ± 2.19	
Grade			=0.110
1-2	34	20.05 ± 2.44	
3-4	4	18.17 ± 1.98	
Stage			=0.004^*^
I-II	6	17.13 ± 2.61	
III-IV	31	20.29 ± 2.20	
T			=0.922
1-2	24	19.42 ± 2.78	
3-4	14	19.52 ± 3.23	

* Statistical significance *P*<0.05.

### CXCL3 promotes HNSCC cell proliferation and invasion

**Figure 7:**
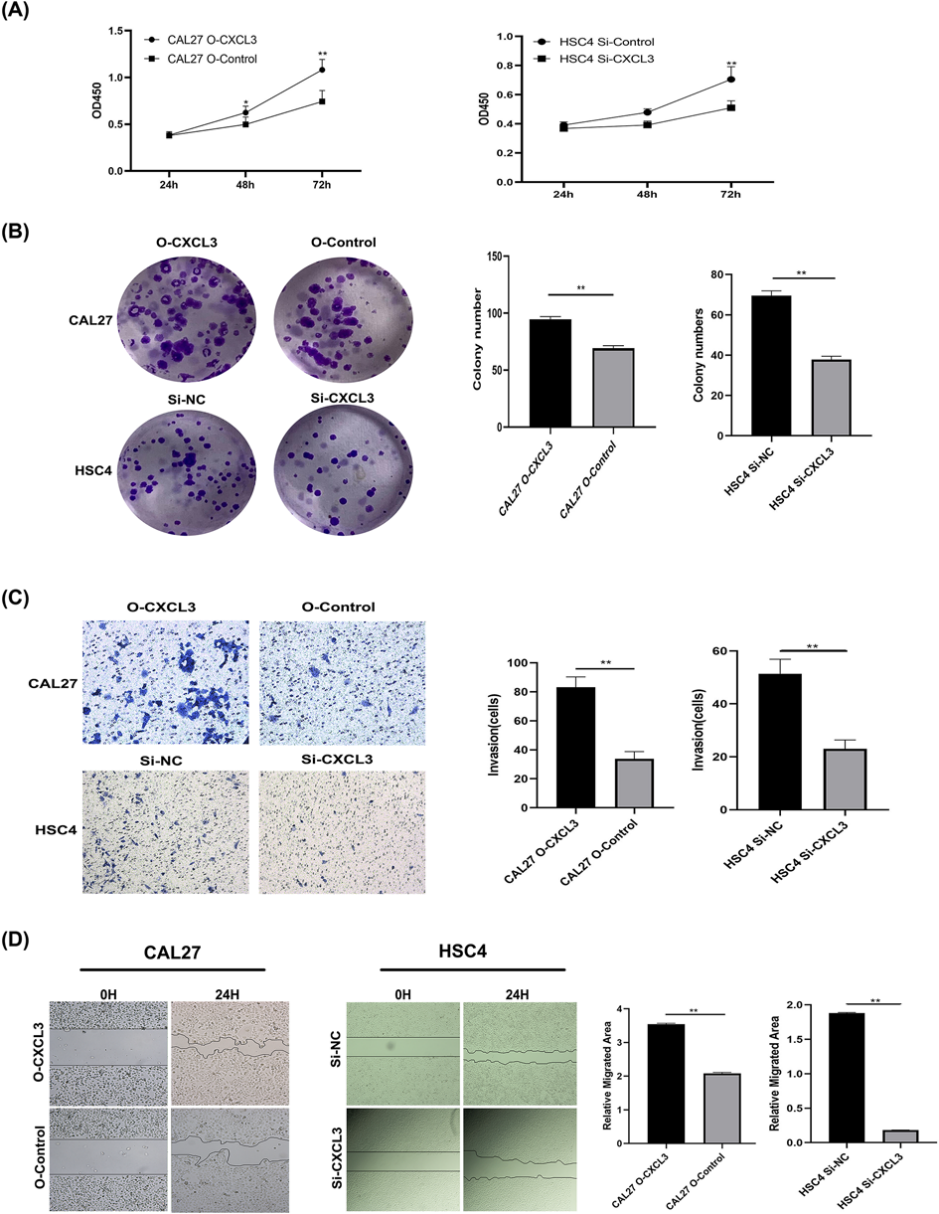
The roles of CXCL3 in HNSCC cell malignant behaviors *in vitro* (**A**) Representative boxplot of CCK‐8 analysis (**B**) Representative images and boxplot of clonogenic analysis. (**C**) Representative images and boxplot of Transwell analysis. (**D**) Representative images and boxplot of wound healing assay. *P<0.05; **P<0.01; HNSCC, head and neck squamous cell carcinoma.

To explore the role of endogenous CXCL3 in cell proliferation and invasion, we established CAL27 cells overexpressing CXCL3 and HSC4 cells downexpressing CXCL3, and then verified the transfection efficiency by qRT-PCR (Supplementary Figure S1). CCK8 was used to detect the role of CXCL3 in the proliferation of HNSCC cells, and the results showed that up-expression of CXCL3 promotes the proliferation of CAL27 cells, while down-expression of CXCL3 significantly inhibits the proliferation of HSC-4 cells (Figure 7A). Furthermore, Clonogenic ability assays showed that CXCL3 over-expression significantly promoted the clonality of CAL27 cells, while CXCL3 down-expression significantly inhibited the clonality of HSC4 cells (Figure 7B). Transwell assay and wound healing assay results also verified that up- and down-expression of CXCL3 significantly promoted and inhibited invision and migration activities of CAL27 and HSC4 cells, respectively (Figure 7C,D).

### CXCL3 inhibits cell apoptosis and regulates the expression of tumor-related proteins

**Figure 8:**
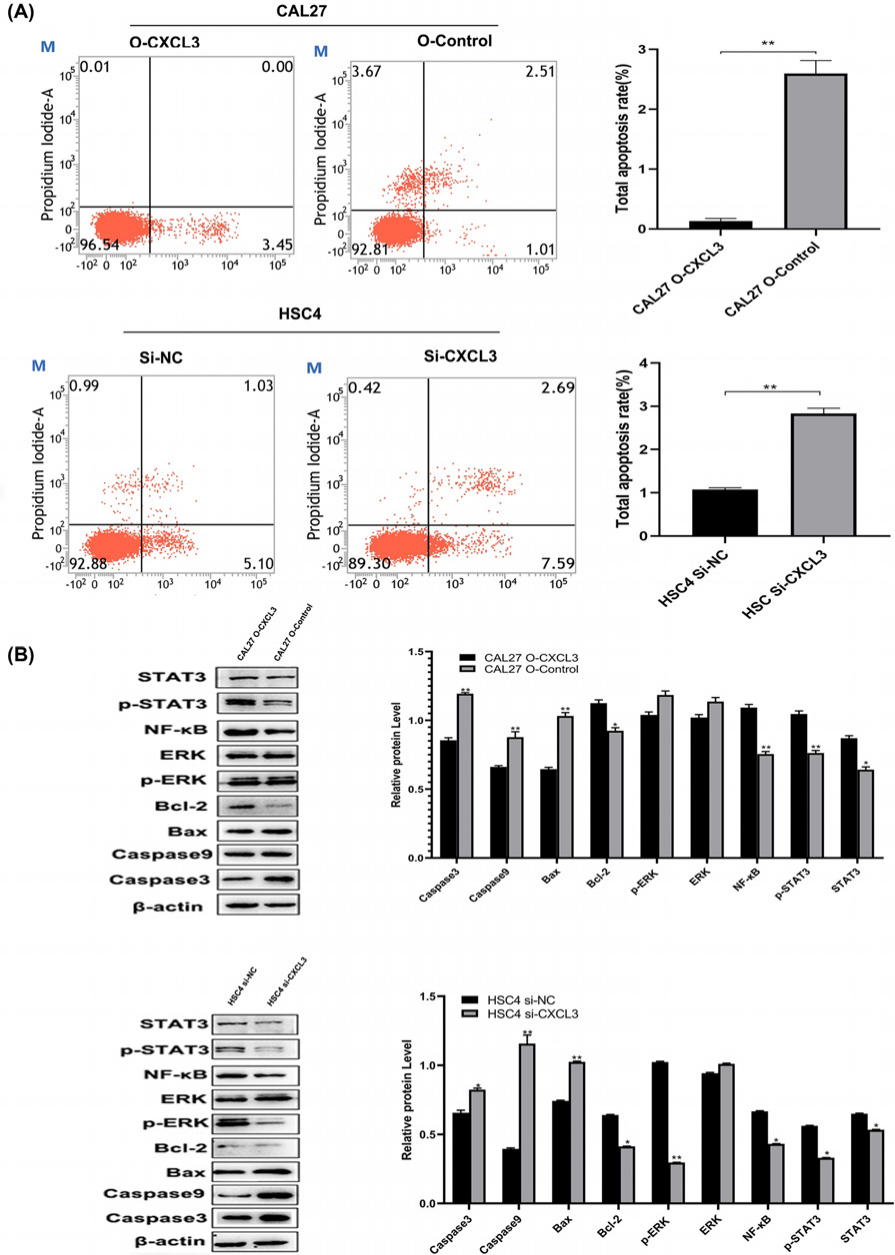
CXCL3 inhibits apoptosis of HNSCC cells and affects expression of tumor-associated proteins (**A**) The apoptosis rate of HNSCC cells were detected by flow cytometry. (**B**) The level expression of proteins in the CXCL3-associated signaling pathways were determined by Western blotting assay. *P<0.05; **P<0.01; HNSCC, head and neck squamous cell carcinoma.

Cell apoptosis were further detected by flow cytometry trying to seek the oncogenic activity of CXCL3 in HNSCC. The result showed that the cell apoptosis rate was significantly decreased after the up-expression of CXCL3 gene in CAL27 cells, whereas the apoptosis rate of HSC4 cells was notably increased when downregulated CXCL3 expression (Figure 8A). To verify the signaling pathways predicted by GSEA, the relevant proteins in these signaling pathways were determined by Western blotting assays. The result showed that CXCL3 up-expression in CAL27 cells increased expression of STAT3, p-STAT3, NF-κB, Bcl-2, and decreased expression of Bax, Caspase9, and Caspase3 whereas down-expression CXCL3 in HSC4 cells significantly decreased the expression of STAT3, p-STAT3, NF-κB, p-ERK, Bcl-2, and increased the expression of Bax, Caspase9, and Caspase3 (Figure 8B).

## Discussion

CXCL3, a single-stranded protein with a relative molecular mass of 8–12 kD, belongs to the family of CXC chemokines [5,12]. Accumulating evidence presented that a variety of cell types, such as, macrophages and neutrophils can produce CXCL3, which in a homeostatic or inflammatory condition has the ability to directly recruit and activate cells expressing CXC-chemokine receptor (CXCR) 2 through autocrine or paracrine fashion [13]. After interacting with its cell surface receptor CXCR2, CXCL3 is participated in regulating numerous physiological and pathological processes, such as chemotaxis, angiogenesis, pregnancy-related diseases, tumors, cardiovascular diseases and lung diseases by regulating the processes of cell migration, invasion, angiogenesis, and fibrosis [14–18]. Many studies showed that CXCL3 is highly expressed in many different types of cancers, including breast cancer, prostate cancer, cervical cancer, and ovarian cancer, and its up-regulated expression is closely related to clinical characteristics. In hepatocellular carcinoma, the up-regulation of CXCL3 expression is associated with a poor prognosis [19]. Bieche et al. [20] exhibited that the relative mRNA expression of CXCL3 was higher in the tissues of grade I invasive breast cancer, grade III invasive breast cancer, and distal metastatic breast cancer than in the normal breast cancer, and then the relative mRNA expression of CXCL3 was the highest in distal metastatic breast cancer. It was demonstrated that CXCL3 is closely related to tumor metastasis of breast cancer [17]. Our previous studies also showed that CXCL3, while being less expressed in normal cervix tissues, is strongly expressed in cervix cancer tissues, of which metastatic cervix cancer tissues had the highest expression of CXCL3 [21].

In the present study, we found that the copy number of the CXCL3 gene in HNSCC tissues increased, and the main types of CXCL3 gene alteration were mRNA High, amplification, and missense mutation, suggesting that CXCL3 expression may be related to the poor prognosis of patients with HNSCC. In agreement with this, a previous study speculated that a polymorphism at the mutation site of the CXCL3 gene may play an important role in tumorigenesis [22]. Subsequently, an analysis of transcriptional sequencing data for more than 500 clinical samples in multiple public databases showed that the expression level of the CXCL3 gene in HNSCC patients, compared with normal tissues, was significantly higher, and our immunohistochemical experiments from clinical samples also confirmed this result. Kaplan–Meier survival analysis showed that African-American HNSCC patients with high expression of CXCL3 had shorter overall survival (*P*<0.05). Univariate COX analysis and multivariate Cox analysis further confirmed that CXCL3 can be used as an independent prognostic factor for overall survival of patients with HNSCC. In addition, our IHC result also showed that the expression of CXCL3 is associated with HNSCC stage. Due to a variety of important physiological functions of CXCL3, abnormal expression of CXCL3 may cause changes in the corresponding regulatory network. We further conducted GSEA analysis to explore CXCL3 associated important target gene functional networks, protein kinases, and transcription factors. GO terms showed that the associate gene network near CXCL3 mainly involves leukocyte migration, endoplasmic reticulum lumen, cytokine receptor binding, and G-protein–coupled receptor binding. These conclusions were consistent with the physiological functions of CXCL3, that is, as a tumor-inducing factor, CXCL3 can promote the chemotaxis and angiogenesis process of a variety of cells, activate G-protein–coupled receptor, induce calcium mobilization and ERK phosphorylation, thus participate in tumor development [23–27]. Understanding the changes in CXCL3 expression can cause severe dysfunction, and even lead to cancers such as HNSCC, requires further research.

Apart from cancer cells themselves, neighboring cells, including stromal and inflammatory cells, are implicated in the secretion of chemokines, which serve as a source of paracrine signals for the proliferation of cancer cells [28]. As the concentration changes, the directional migration ability of chemokines also changes [29]. In our previous research, we found that CXCL3 affects the biological behavior of tumor cells in the form of autocrine or paracrine [8]. We also confirmed this conclusion by giving exogenous CXCL3.

In the present study, we indicate that up-regulating the expression of CXCL3 in HNSCC cells helps to enhance the malignant behaviors of HNSCC cells, including proliferation, colony formation, and migration. Whereas these malignant behaviors of HNSCC cells were decreased after down-regulating CXCL3 expression At the same time, we found that the high CXCL3 expression was associated with apoptosis, Toll-like receptor signaling pathway, Nod-like receptor signaling pathway, Jak-STAT signaling pathway, and MAPK signaling pathway by GSEA. The biological effects of Toll-like receptor signaling pathway activation are mainly reflected in the production of a variety of cytokines and DC activation [30]. The abnormal regulation of this signaling pathway may lead to inflammation, excessive repair, and tumor formation [31]. NOD-like receptors are a type of pattern recognition receptor of the host, which can induce the production of IL-1β and IL-18 by regulating the formation of inflammasomes, thereby participating in the inflammatory response [32–33]. When the Jak-STAT signal pathway is abnormally activated, the stability of the genome decreases, and the cell cycle becomes abnormal, leading to tumor formation [34–35]. Themitogen-activated protein kinase (MAPK) cascade is a key signaling pathway that regulates cell proliferation, cell differentiation, cell apoptosis, and stress response [36]. ERK expression is essential for development, and their over-activation plays a major role in the development and progression of cancer [37]. In the present study, we finally, using Western blot experiments, identified that overexpression of CXCL3 in HNSCC cells regulate the expression of several important proteins including Bax, Bcl-2, p-ERK, NF-κB, p-STAT3, and STAT3 in above-mentioned pathways. Lin et al. study has shown that CXCL3 may participate in the feedback regulation of CD133 expression in liver cancer through the MAPK/EST1 pathway, and patients with high expression of CXCL3 showed poor prognosis [19]. In a large-scale loss-of-function screen, Lauren et al. found that CXCL3 promoted STAT3 activation in CD44+CD24-human breast cancer cells. CXCL3 promoted breast cancer cell proliferation through the JAK2/STAT3 signaling pathway [38].

In summary, the present study illustrated that CXCL3 is highly expressed in HNSCC, and its up-regulated expression is closely related to the poor clinical characteristics of HNSCC, thus can be served as an adverse independent prognostic factor for patients with HNSCC. Our findings also revealed that CXCL3 contribute to the malignant behaviors of HNSCC cells through several tumor related signaling pathways including Toll-like receptor, Nod-like receptor, Jak-STAT and MAPK signaling pathways, suggesting that CXCL3 may play a crucial role in the occurrence and development of HNSCC.

## Supplementary Material

Supplementary Figures S1-S2

## Data Availability

The datasets used and/or analyzed during the current study are available from the corresponding author on reasonable request.

## Abbreviations

ERK: extracellular signal-regulated kinase
FBS: fetal bovine serum
HNSCC: head and neck squamous cell carcinoma
HR: hazard ratio
PBS: phosphate-buffered saline
HRP: Horseradish Peroxidase
MAPK: mitogen-activated protein kinases
STAT3: Signal transducer and activator of transcription 3
