# Supplementary material for: Clinical significance and biological functions of chemokine CXCL3 in head and neck squamous cell carcinoma
Source: Biosci Rep. 2021 Dec 22;41(12):BSR20212403. doi: 10.1042/BSR20212403 (PMC8696619; doi:10.1042/BSR20212403)
Supplement: Supplementary Figures S1-S2 [file BSR-2021-2403_supp.pdf]

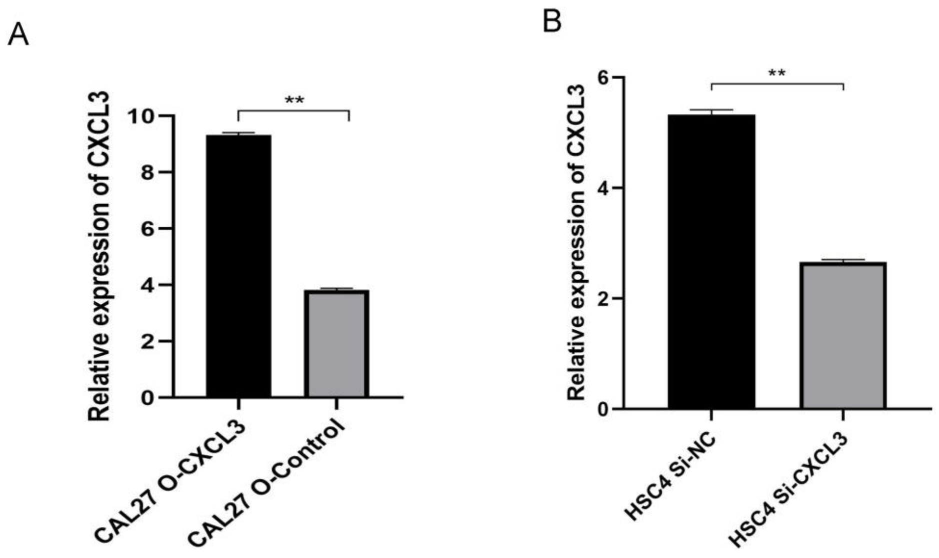

Figure S1 CXCL3 mRNA expression levels in HNSCC cell were detected by qRT-PCR. (A) Relative mRNA expression of CXCL3 in CAL27 cells. (B) Relative mRNA expression of CXCL3 in HSC4 cells.

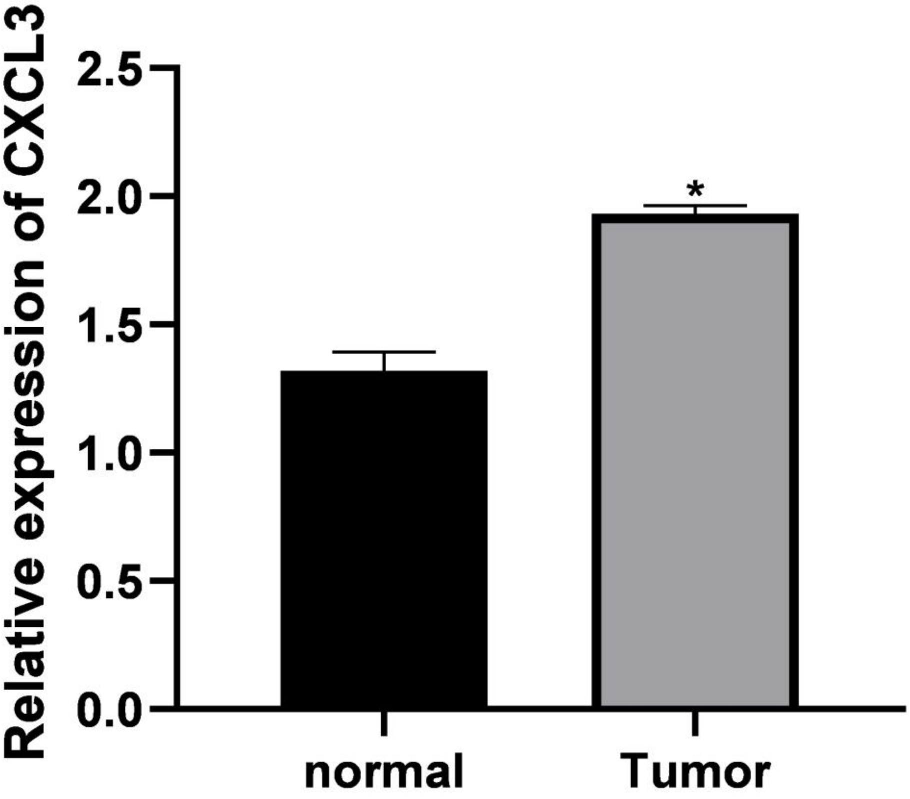

Figure S2 The expression level of CXCL3 in serum samples from the GEO database.
